# Creating chronicity

**DOI:** 10.1111/jep.12715

**Published:** 2017-05-12

**Authors:** Anna Luise Kirkengen

**Affiliations:** ^1^ General Practice Research Unit, Department of Public Health and General Practice Norwegian University of Science and Technology Trondheim Norway; ^2^ Department of Community Medicine UiT The Arctic University Tromsø Tromsø Norway

**Keywords:** causality, multimorbidity, person‐centered medicine

## Abstract

An authentic sickness history is the vantage point for juxtaposing a biomedical and a biographical‐phenomenological reading. What, in a biomedical framework, appears to be a longstanding state of comorbidity of different and unrelated types of diseases is rendered transparent in a biographical reading.

This particular reading, evidencing the shortcomings of a biomedical framework regarding identifying the social sources of an increasingly complex burden of disease, is reflected upon in light of recent research in the neurosciences. Thus, the biomedical contribution to a sickness history is demonstrated, with its resultant multimorbidity, chronification, and complete incapacitation of a woman despite the continuing and nearly excessive involvement of the health care system.

## 1 INTRODUCTION

Departing from the increasingly appraised methodological concept of N *=* 1, this paper represents a voice in the current medicoprofessional discourse concerning the appropriateness of departing from “only” 1 case, patient or sickness story; thus, challenging the methodological presupposition of the “anecdotal” as a nonscientific and nonreliable source of knowledge.

This paper also includes a reflection upon the ontological premises for a particular workshop's subheading, *Causal reasoning and evidence for clinical decisions* *
CauseHealth Workshop titled N = 1—Causal Reasoning and Evidence for Clinical Practice, January 12th, 2016, NMBU Campus, Ås, Norway. taking it literally or even personally. The paper presents an authentic life and sickness history, in other words, the story of a person thus adhering to the quest and central stance of phenomenology, namely, to interrogate, or rather literally go “zu den Sachen selbst,” in this particular context “zu der Sache selbst” in the sense of appraising the voice of a chronically diseased person. This particular person's voice and her own biographical accounts will be juxtaposed with the biological accounts in the voice of biomedicine. In this process, the notion of “cause” is implicit, engendering the question or whether and, if so, how, the narrative about lived life, offered in the first person voice, might be both appropriately and fruitfully integrated with the knowledge fund about human pathophysiology, presented in the third person voice, the voice of biomedicine. A possible answer to this question might be found embedded in a proposal by Kerry et al regarding a dispositional ontology as a more appropriate guide for medical causal reasoning than the prevailing approach.^1^

## 2 THE CASE

The main referential framework for juxtaposing condensates of authentic first and third person accounts is a sickness history that—in the current biomedical framework—is identified as a history of chronic, generalized pain in a nearly 60‐year‐old woman, Berit Bruun (pseudonym), †
The person has given her consent to the current publication. who has been exposed to a variety of treatments without satisfactory effect. She has been given, among many others, the diagnosis of Fibromyalgia. Earlier in her sickness “career,” in the course of various treatments over time, she became addicted to specific analgesic drugs, an addiction she strived for years to overcome. At present, she is using both analgesic and anti‐inflammatory drugs on a regular basis in high‐daily dosages.

A few years ago, when attending the regional outpatient pain clinic after months on the waiting list, the first treatment—injections to block the transport of pain impulses in certain regions—failed. Next, acupuncture proved moderately effective, engendering the assumption that she might benefit from having a TENS machine available at home (providing a kind of electric acupuncture without needles), enabling her to administer a procedure for self‐treatment in case the pain became intolerable. Berit herself experienced this “machine‐inherent” independence from outpatient clinic appointments and the varying availability of professionals as the most remarkable and liberating aspect of this kind of medical treatment.

In addition to chronic pain, Berit has suffered from, and is still affected by, what is conceptualized as longstanding eating problems. Initially diagnosed as anorexia, this later turned into bulimia, resulting during the last decades in an ever increasing and now gross obesity, a constant source of critical and humiliating comments from her family, and a deep source of shame. She knows that her body is “read” as attesting to her lack of will power, self‐discipline, or impulse control. Therefore, during various periods of her life, she has tried to regulate her weight by using both laxatives and diuretic medications, although without resulting in weight loss.

As a long‐term consequence of obesity, she has been subjected to necessary surgical interventions on both her hips and knees to compensate for increasing deformity. A few years ago, she underwent gastric surgery to achieve weight reduction since no other measures had sufficient effect. The surgical intervention, performed without complications, was deemed highly successful as it resulted during the following year in a weight loss equaling more than a third of Berit's previous body volume. The highly problematic impact of this success was the surplus of skin remaining in huge folds on, for example, her arms and thighs, abdomen, and buttocks. These folds collected moisture, which promoted the growth of fungus, causing rashes, itching, localized inflammation, and, not least, causing Berit great discomfort when both walking and sitting. All these health problems necessitated surgical skin reduction. Her arms and thighs were sculpted first. Although the next plastic surgery procedure, an abdominal correction, was performed without complications, there resulted nevertheless increasing intra‐abdominal pain, suspected to indicate a technical mistake having been made during the intervention. When the pain continued unabated, abdominal endoscopy was deemed necessary. During this procedure, a highly unexpected complication, an intestinal perforation, caused a crisis for the patient. She lost consciousness and could not be reawakened from anaesthesia, necessitating several weeks of close surveillance at the intensive care unit of the hospital. After recovery, a range of chronic health problems remained, such as nausea, frequent vomiting, complex pain, loss of memory, nightmares, problems with concentration and sleep, and eating problems. This nexus of symptoms, reminiscent of a clinical picture termed post‐traumatic stress disorder, made Berit's everyday life quite demanding. In addition, because of the aforementioned complications, the next scheduled plastic surgery intervention to correct the skin folds on her buttocks, which troubled Berit both while seated and when walking, had to be permanently cancelled.

## 3 THE PERSON

Berit Bruun herself dates her childhood as the start of her eating disorders and subsequent weight problems. She describes herself as constantly tip‐toeing, with her head drooping between her shoulders. This is how she portrays her bodily habitus both as a child and, even more explicitly, as an adolescent. By now, she has acknowledged that she has been on her guard since early childhood, restricting herself and adapting to others' more or less explicit demands, which has informed her bodily being.

Since childhood, she has been under the consistently harsh control of her violent father. Preoccupied with keeping his daughter untouched by males in general, he guarded her against presumed dangers from outside, while remaining unaware that the girl was being exploited and abused by her elder brother in the family's home. The brother made her stand guard during his nightly acts of petty criminality and serve his sexual demands, night after night, from when she was in primary school until she was 18 years old and fled from the home where she had no place to hide or to rest. She moved in with the family of a former classmate, who she married shortly afterwards, becoming aware almost immediately that this marriage was a mistake. However, being an unskilled worker with a limited ability to earn her own living, she saw no other option than to marry as soon as possible. Since her parents were apparently unaware of what her brother did to her, he had abused her as he pleased. She was too afraid to object or resist her physically well‐trained brother, who also battered her whenever he pleased.

Early in her teens, Berit had anxiety attacks and cramps, predominantly in her hands and forearms. Suspected of having epilepsy, she was examined by specialists in child neurology. As no neurological defect could be demonstrated, they concluded that her cramps were of psychogenic origin. However, the specialist did not ask her whether she herself had any assumptions as to why she had these so‐called pseudoseizures in her hands and forearms. Had they asked, she might have been able to identify a connection to her being forced by her brother, night after night, to masturbate him, after his having penetrated her body's openings with a variety of instruments.

Soon after this examination, Berit started cutting herself seriously. She was stitched countless times at the emergency ward in her hometown. Nobody asked her what she wanted to cut off or whom she actually wanted to attack, had she dared, instead of attacking herself. Had one of the various professionals she encountered asked in an open‐minded and empathetic way, she might have dared to recount that several people used such a degree of force on her that she could not stand it anymore.

At 17, she made her first attempt to commit suicide and was admitted to a psychiatric ward. Nobody asked her why she did not want to live. Had somebody asked, carefully and insistently, she might have identified the persons who made life unbearable for her. During a much later admission to a psychiatric ward, Berit finally found the courage to inform a psychologist. She was supported in her wish to confront her family with the years of abuse, maltreatment, neglect, and exploitation. She was 30 years old by then. Her parents and brothers seemed to listen to her accusations; her elder brother even admitted having had sexual contact with her but described it as playing a game in which she had participated voluntarily.

Again, Berit felt abandoned by her entire family. In retrospect, she can see that she was far too scared to be clear and insistent about her accusations. In addition, as if she had not spoken at all, her father and brother continued to treat her with scorn and disrespect. Her brother even exploited her economically, which Berit felt forced to keep concealed from her husband. At this point, she is fully incapacitated because of chronic, widespread, nonmalignant pain, anxiety, a panic disorder, eating disorders, and gross obesity. She also reports that her father despises no one as much as those who cannot make their own living.

## 4 OBJECTIFIED

In biomedical terms, Berit's chronic pain is categorized as being of muscular or/and skeletal origin. She is diagnosed as having fibromyalgia syndrome (FMS), a contested diagnosis regarding how it arises, what it means, and how the condition is to be treated. Means to objectify the pain are sought since reports of pain are considered as subjective accounts in biomedicine, and, as such, as invalid or unreliable data. Barker has analysed and described how, in the case of FMS, rheumatologists constructed a set of diagnostic criteria involving tender trigger points, painful areas mapped on the body and “objectified” by means of a pain response to a certain thumb pressure applied by the physician in a systematic way.^2^ The evocation of pain through the application of pressure is considered an objective finding and, therefore, valid. Thus, thumb pressure gained instrumental status by “transforming” a reported pain into a proven pain, rendering the pain an objective entity that could be dealt with as if it were not subjective (although it still is). Thus, it is real. The real pain is proof of a real disease, defined as being in the realm of rheumatology, although no inflammation or autoimmune origin of the pain has been demonstrated. The making of FMS rests on a scientific flaw: the tender points both define and substantiate the diagnosis. Epidemiological data about FMS are constituted by tautologies. In addition, research has solidly documented a comprehensive “overlap” of chronic pain with a variety of other diagnoses—so‐called multimorbidities or comorbidities. This means people “in pain” are also otherwise “in trouble.”^3^

When applied to Berit's life and sickness history, judged not from a biomedical perspective but interpreted within an existential perspective, the typical relational and social phenomena informing both her life and her self‐esteem may be characterized by a series of negatively charged words. These denote conditions or situations that engender sickness if not buffered by good relationships or supportive experiences.^4^ Her history is replete with violence, fear, threats, abuse, duties, harassment, defeats, control, neglect, abandonment, obedience, exploitation, anxiety, subordination, scorn, surveillance, disrespect, and powerlessness.

These words signify objectification and “thingification.” All her life she has been the object of others' disregard, scorn, and disrespect. Incessantly, she has been abused, exploited, and invaded. Her attempts to demarcate a boundary and protect herself have been jeopardized, ridiculed, and responded to with the opposite: violation of her integrity, again and again. Thus, something has happened that comes with “Being Made a Thing”: she has internalized others' view of her as accurate, as the real Berit.^5^ She has come to identify with the ugly, stupid, and unworthy thing her family says that she is and that is shameful.

Being exposed from early childhood to what is now termed “toxic” stress, because it has been unabated and not buffered,^4^ her physiology has been radically disturbed in what researchers in the neurosciences now model as “multisystem physiological dysregulation.^6^ This implies that the most primary guardians of viability and adaptability, namely, the hormonal, immune, and central nervous systems, including the functions exercised by the autonomous nervous system, and the metabolism of lipids, glucose, and minerals, are all disturbed, both separately and in their multilayered interplay. In clinical findings, such multisystem dysregulation typically results in complex patterns of so‐called multimorbidity, comprising various states of diseases currently diagnosed as somatic, psychiatric, so‐called functional, or medically unexplained. These states of complex sickness engender yet another health risk, namely, that which is inherent in the highly unpredictable, accumulated impact of drugs which, evaluated separately, would seem appropriate for each of the diagnoses.

As to the impact of longstanding toxic stress on the unfolding structure of a child's brain, this documentation has recently been reviewed by Teicher and Samson.^7^ These researchers conclude that the inscriptions of early abuse, maltreatment, neglect, and deprivation on the brain can be evaluated in not only their effect on form but also function and connectivity. In other words, all levels of the brain are adversely affected. Also, and most decisively, the researchers conclude “Structural and functional abnormalities initially attributed to psychiatric illness may be a more direct consequence of abuse. Childhood maltreatment exerts a prepotent influence on brain development and has been an unrecognized confound in almost all psychiatric neuroimaging studies. These brain changes may be best understood as adaptive responses to facilitate survival and reproduction in the face of adversity”.^7^ Further on, the authors delineate the direct impact of various types of sensory perceptions on the brain structures involved in their interpretation, thus being bodily inscriptions in a most literal sense. These results, from studies based on the most advanced visualizing techniques, confirm the descriptions of such relationships based on personal accounts as related in in‐depth interviews with sexually molested persons.^8^

## 5 IN CONCLUSION

The scientific community has, by now, acknowledged that social disadvantage engenders pathophysiology. This means that the causes of health and sickness are grounded in the interplay between person and context, and with biology and biography intertwined. This means also that causality, as in belonging to the physical world, and reason, as in belonging to the social world, are also intertwined. Consequently, the traditional scientific concept of causality simply does not apply to human health and sickness. In other words, and to quote Kerry et al: “The greatest causal work can be seen in single‐instance cases. This is where the real nature of causation is witnessed.” Thus, I claim that unless a case is acknowledged as regarding a person embodying her or his life and adequately enriched with that person's account of relevant life course information, the risk is high that the sufferer will be misinterpreted. It follows logically that this leads next to a high probability of diagnostic and therapeutic mistakes appearing as so‐called multimorbidity, resulting in dangerous and potentially fatal polypharmacy, as well as to medically induced chronification, and to complete incapacitation.

## References

[1] Kerry R , Eriksen TE , Lie SAN , Mumford SD , Anjum RL . Causation and evidence‐based practice: an ontological review. J Eval Clin Pract. 2012;18:1006–1012. 22994999 10.1111/j.1365-2753.2012.01908.x

[2] Barker KK . The Fibromyalgia Story. Medical Authority and Women's Worlds of Pain. Philadelphia: Temple University Press; 2005.

[3] Eriksen TE , Kirkengen AL , Vetlesen AJ . The medically unexplained revisited. Med Health Care Philos. 2013;16:587–600. 23054425 10.1007/s11019-012-9436-2

[4] Shonkoff JP , Boyce WT , McEwen BS . Neuroscience, molecular biology, and the childhood roots of health disparities. Building a new framework for health promotion and disease prevention. JAMA. 2009;301:2252–2259. 19491187 10.1001/jama.2009.754

[5] Jones CP . Levels of racism: a theoretical framework and a gardener's tale. Am J Public Health. 2000;90:1212–1215. 10936998 10.2105/ajph.90.8.1212 PMC1446334

[6] Wiley JF , Gruenewald TL , Karlamangla AS , Seeman TE . Modeling ultisystem physiological dysregulation. Psychosom Med. 2016;78:290–301. 26734956 10.1097/PSY.0000000000000288 PMC4844860

[7] Teicher MH , Samson JA . Annual research review: enduring neurobiological effects of childhood abuse and neglect. J Child Psychol Psychiatry. 2016;57(3):241–266. 26831814 10.1111/jcpp.12507 PMC4760853

[8] Kirkengen AL . Inscribed Bodies. Dordrecht: Kluwer Academic Publishers (now Springer); 2001.

